# Motor Ability Evaluation of the Upper Extremity with Point-To-Point Training Movement Based on End-Effector Robot-Assisted Training System

**DOI:** 10.1155/2022/1939844

**Published:** 2022-01-28

**Authors:** Junwei Jiang, Shuai Guo, Leigang Zhang, Qing Sun

**Affiliations:** ^1^School of Mechatronic Engineering and Automation, Shanghai University, Shanghai 200444, China; ^2^National Demonstration Center for Experimental Engineering Training Education, Shanghai University, Shanghai 200444, China

## Abstract

Assessment is critical during the procedure of stroke rehabilitation. However, traditional assessment methods are time-consuming, laborious, and dependent on the skillfulness of the therapist. Moreover, they cannot distinguish whether the improvement comes from the abnormal compensation or the improvement of upper extremity motor function. To make up for the shortcomings of the traditional methods, this study proposes a novel assessment system, which consisted of a rehabilitation robot and motion capture (MoCAP) system. A 9-degree-of-freedom (DOF) kinematic model is established, which consists of the shoulder girdle, shoulder, elbow, and wrist joints. And seven assessment indices are selected for this assessment system, including a range of motion (ROM), shoulder girdle compensation (SGC), trunk compensation (TC), aiming angle (AA), motion error (ME), motion length ratio (MLR), and useful force (UF). For AA, ME, and MLR, all describe the motor ability of the upper extremity, and a linear model was proposed to map these three indices into one index, called motor control ability (MCA). Then, this system can quantitatively evaluate human upper extremity motor function from joint space kinematics, Cartesian space kinematics, and dynamics. Three healthy participants were invited to verify the effectiveness of this system. The preliminary results show that all participants' handedness performs a little better than the nonhandedness. And the performance of the participants and the change of all the upper limb joints can be directly watched from the trajectory of the hand and joint angles' curve. Therefore, this assessment system can evaluate the human upper limb motor function well. Future studies are planned to recruit elderly volunteers or stroke patients to further verify the effectiveness of this system.

## 1. Introduction

Stroke is the second highest cause of death globally [1]. It usually has a high rate of disability, and stroke patients are usually accompanied by upper limb dyskinesia, abnormal muscle tone, and decrease in somatosensation, which seriously influence their daily life [2]. Thus, a systematic and effective assessment of the human upper extremity function is extremely important for the next stage of clinical decision making [2, 3]. The traditional assessment method is according to the clinical scales, such as Fugl-Meyer Assessment (FMA) [4] and Motor Power (MP) clinical impairment scales. However, the traditional methods are time-consuming, laborious, and dependent on the skillfulness of the therapist [5]. Moreover, they cannot distinguish whether the improvement comes from the abnormal compensation or the progress of upper extremity motor function [6, 7]. To make up for the shortcomings of traditional methods, more and more scholars established the assessment system to quantitatively evaluate the upper extremity motor function by combining robot and sensors [8–10] or only using sensors, such as Kinect [11] and MCU [12].

Kinematics can provide more accurate real-time indicators of patients' recovery as compared with the traditional assessment method, which describes the movements of the body through space and time, including linear and angular displacements, velocities, and accelerations, and can provide a better understanding of human movement [13]. Bosecker et al. [14] analyzed the Cartesian space kinematics of the hand and established a linear regression model to quantitatively assess the upper extremity function by InMotion2. Zollo et al. [15] used a magnetic inertial sensor to collect the acceleration of the hand and performed submotion decomposition to evaluate the human upper extremity function. Coderre et al. [16] evaluated the Cartesian space kinematics of hand in the target reaching task to assess the upper extremity motor function by an exoskeleton dual-arm robot, KINARM. Although these scholars analyzed the Cartesian space kinematics to replace the traditional assessment method, they still cannot show which joint recovers better or less. Therefore, many scholars use the optical motion capture (MoCAP) system to collect upper extremity joint space kinematic information and apply them as indicators for the assessment of upper extremity function.

Murgia [17] established a 7-degree-of-freedom (DOF) kinematic model, which could obtain the upper extremity joint space kinematic information and also could distinguish the trunk compensation, to evaluate the upper motion function in the activity of daily living (ADL) by MoCAP system. Murphy et al. [18] obtained the flexion/extension angles and abduction/adduction angles of the shoulder joint and the flexion/extension angles of the wrist joint during the drinking water exercise and discussed the interjoint coordination between the shoulder and elbow joint. To quantitatively assess upper extremity function, Hebert et al. [19] analyzed the DOFs of the shoulder, elbow, and wrist joint during the box and blocks task. Although these authors analyzed the upper extremity joint space kinematics by the MoCAP system to assess the upper extremity motor ability, they ignored the compensation motion, which could not distinguish real restitution from abnormal compensation exactly.

This paper proposes a quantitative evaluation system for human upper extremity motion ability and builds a comprehensive 9-DOF model on the human upper extremity, which is based on robot and the MoCAP system. And our assessment system contains the joint space kinematics, Cartesian space kinematics, and dynamics indices. It can not only assess the function of the patient's upper extremity well but also recognize the compensation of the shoulder girdle and trunk to distinguish whether the improvement is due to abnormal compensation or improvement in motor ability.

The remainder of the paper is arranged as follows: firstly, the description of the mechanical structure of the end-effector robot-assisted training system, a novel 9*-*DOF upper extremity model, assessment indices, and the linear model are presented, respectively, in Section 2; three healthy subjects were recruited to verify and evaluate the effectiveness of our system, and the results are shown in Section 3; in Section 4, we discuss the results of the experiment and compare the differences between our evaluation system and others. Conclusions and further works are drawn in Section 5.

## 2. Methodology

### 2.1. Notations

Table 1 shows the abbreviations used in this paper.

### 2.2. Description of the Evaluation System

**Figure 1:**
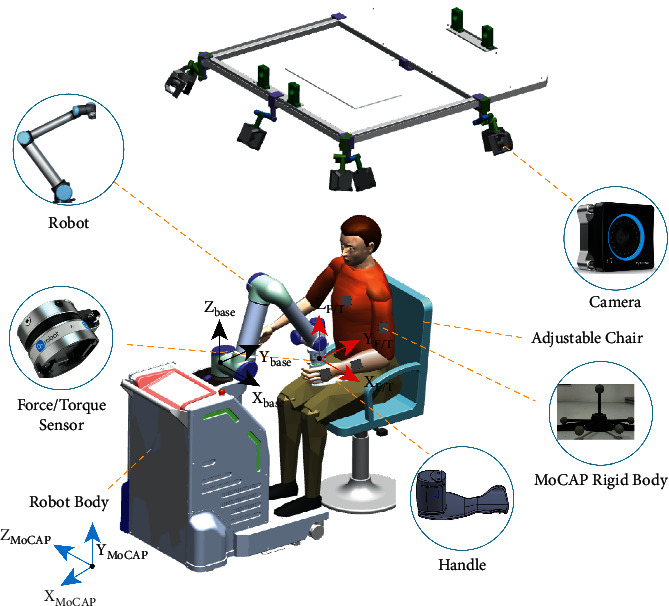
Schematic diagram of evaluation system structure.

The evaluation system consists of an end-effector-robot-assisted system and MoCAP system. The overall structure of this system is shown in Figure 1. The robot body structure module, which is equipped with six universal wheels, is mainly used to support the assistance motion module, as well as the robot control cabinet, the control computer, and the electrical structure. And the robot system can provide planar or three-dimensional space motion and active or passive motion. In this paper, the active motion based on the horizontal plane is selected to evaluate the function of the human upper extremity. The robot system can provide the information of Cartesian space kinematics relative to the base coordinate system of the robot in real-time. And there is a 6-axis force/torque sensor (Hex) at the end effector, which is used to obtain the information of the patient's force and torque in real-time. All the base coordinate is the same as our previous study [20], as shown in Figure 1. The control principle of the robot is based on the admittance control:

(1) $$\begin{array}{c} M\Delta \ddot{x}+D\Delta \dot{x}+K\Delta x=f_{e}, \end{array}$$

where *M* represents the inertia characteristic matrix, *D* represents the damping characteristic matrix, *K* represents the stiffness characteristic matrix, and *f*_*e*_ is the external force provided by the robot.

The joint space kinematics of the upper limb are recorded by a 3D MoCAP system consisting of several optical cameras. Five MoCAP rigid bodies are used to capture the joint space kinematic data of human upper extremity. And the position and pose of rigid body are captured by each motion camera. Besides, the base coordinate frame of the optical MoCAP system is set as shown in Figure 1. In addition, there is also a movable additional screen for the scene training, placed in front of the patient. It provides visual cues for the patient but is not shown in Figure 1.

### 2.3. Kinematic Model of the Upper Extremity

To build and solve the kinematic model of the human upper limb, we need some known conditions, namely, the position and posture of the joint of the human upper limb. Firstly, according to the theory of rigid body hypothesis, the pose and position of the MoCAP rigid body in the global coordinate system can be transformed into that of upper extremity joint in the global coordinate system.

**Figure 2:**
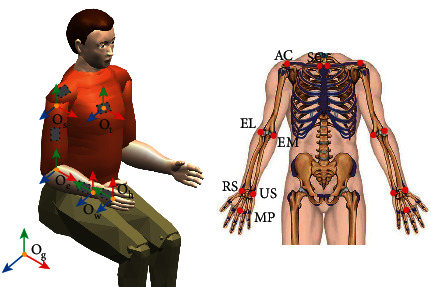
Schematic diagram of static calibration. (a) The coordinate frame of each joint, which is red for the *x*-axis, green for the *y*-axis, and blue for the *z*-axis. The gray rectangle is the MoCAP rigid body. (b) Anatomical landmarks for the static calibration.

Before the transformation, static calibration is needed to determine the joint center and joint coordinate system of each joint. The joint center and joint coordinate frame are established by the position information of seven human anatomical landmarks collected by an auxiliary MoCAP rigid body. And these landmarks are sternoclavicular (SC), acromion (AC), lateral condyle (EL), medial condyle (EM), radial styloid process (RS), ulnar styloid process (US), and metacarpal and phalangeal bone (MP). During the calibration, the subjects were asked to keep their upper body upright, their upper arms perpendicular to the floor, their forearms perpendicular to the upper arm, and their palms facing each other, as shown in Figure 2.

After obtaining the information of these anatomical landmarks, the origin and coordinate systems of the upper limb joint can be constructed, as shown in Table 2. And the coordinate system definition for each joint is mainly based on the International Society of Biomechanics (ISB) [21].

The transfer matrix of each joint relative to the MoCAP rigid body is obtained as follows, the same as our previous study [22]:

(2) TJiBi=TBiG−1TJiG, i=1,2,3,…,5,

where _*J*_*i*__^*B*_*i*_^*T* represents the pose matrix of the joint coordinate system relative to the rigid body coordinate frame, _*B*_*i*__^*G*^*T* is the pose matrix of the MoCAP rigid body in the global coordinate system, which can be directly got from the MoCAP system, and _*J*_*i*__^*G*^*T* is the pose matrix of the joint coordinate frame in the global coordinate system, which can be obtained from static calibration.

Then, the pose matrix of the joint at any sampling time in the global coordinate system can be obtained:

(3) TJiG=TBiG·TJiBi.

**Figure 3:**
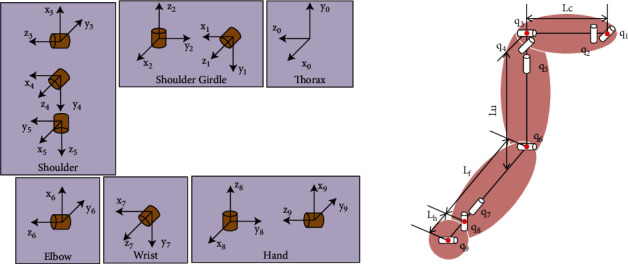
Kinematic model of the right limb. The base reference system is set at the thorax joint. *L*_*c*_ is the distance from AC to SC, *L*_*u*_ is the length of the upper arm, *L*_*f*_ is the length of the forearm, and *L*_*h*_ is the distance between the wrist joint and the hand joint.

When analyzing the motion of the human upper extremity, the upper limb is usually simplified as a 7-DOF [23, 24] or 5-DOF [25, 26] model. However, the human upper limb is extremely flexible and complex, especially the movement of the shoulder joint, which is usually accompanied by the movement of the shoulder girdle leading to the change of the position of the shoulder joint center. Therefore, a 9-DOF simplified kinematic model of the human upper extremity is proposed in this work. Two additional degrees of freedom are used to describe the motion of the shoulder girdle, which not only can detect the motion of shoulder girdle compensation but also allows for a more accurate determination of the origin of the shoulder joint. As shown in Figure 3, the shoulder girdle joint was supposed to be linked to the chest by two revolute joints that contain shoulder girdle elevation/depression (*q*_1_) and profusion/retraction (*q*_2_). Then, the shoulder joint is supposed as a spherical joint, including flexion/extension (*q*_3_), abduction/adduction (*q*_4_) and internal/external rotation (*q*_5_). And the elbow joint is supposed as a hinge joint including flexion/extension (*q*_6_) . Finally, the wrist joint is supposed as a spherical joint, including pronation/supination (*q*_7_), flexion/extension (*q*_8_), and radial/ulnar deviation (*q*_9_).

The first two DOFs of the kinematic model can be solved by the following equations:

(4) $$\begin{array}{c} q_{1}=a\;\cos\left(\frac{sa^{\left(s1\right)}\cdot sa^{\left(s0\right)}}{\left|sa^{\left(s1\right)}\right|\times \left|sa^{\left(s0\right)}\right|}\right), \end{array}$$

(5) $$\begin{array}{c} q_{2}=a\;\cos\left(\frac{sa^{\left(v1\right)}\cdot sa^{\left(v0\right)}}{\left|sa^{\left(v1\right)}\right|\times \left|sa^{\left(v0\right)}\right|}\right), \end{array}$$

where *sa*^(*s*1)^ and *sa*^(*v*1)^ are the projection of the line connecting SC and AC  in the frontal and horizontal plane and *sa*^(*s*0)^ and *sa*^(*v*0)^ are the line connecting SC and AC  in the frontal and horizontal plane at the initial time.

For the last 7 DOFs of the kinematic model, we use the Denavit–Hartenberg convention to analytically describe the kinematic chain. According to the DH rule, the homogeneous transformation matrix between adjacent connecting rods is described as _*i*_^*i* − 1^*T*, which can be calculated by the following equation:

(6) Tii−1=cθi−sθi0ai−1sθicαi−1cθicαi−1−sαi−sαidisθisαi−1cθisαi−1cαicαidi0001,

where *c*_*θ*_*i*__ represents cos *θ*_*i*_, and *s*_*θ*_*i*__ represents sin *θ*_*i*_.

The detailed DH parameters are described in Table 3 and the base coordinate system is located at the shoulder joint center.

According to the DH table, the homogeneous matrix of the upper limb kinematic chain can be obtained by multiplying (6) in orderly, as shown in the following equation:

(7) T93=∏i=39Tii−1,

*q*_3_ and *q*_4_ can be solved according to the transformation matrix of the elbow joint relative to the shoulder joint, namely, _6_^3^*T* as follows:

(8) T63=ox6ax6Px6ny6oy6ay6Py6nz6oz6az6Pz60001,

(9) $$\begin{array}{c} q_{4}=a\;\sin\left(\frac{P_{z}^{\left(6\right)}}{L_{u}}\right), \end{array}$$

where *L*_*u*_ is the length of the upper arm. *q*_6_ can be calculated by the law of cosines:

(10) $$\begin{array}{c} q_{6}=\frac{\pi }{2}-a\;\cos\left(\frac{\left(L_{u}^{2}+L_{f}^{2}-d^{2}\right)}{\left(2^{*}L_{u}^{*}L_{f}\right)}\right), \end{array}$$

where *L*_*u*_ is the length of the upper arm, *L*_*f*_ is the length of the forearm, and *d* is the distance between the shoulder joint and the wrist joint. *q*_5_ can be solved according to the transformation matrix of the wrist joint relative to the shoulder joint, namely, _7_^3^*T*:

(11) q5=a tan 2sin q5, cos q5,cos q5=Px7+Py6+sq3Lfcq4sq6+sq4sq5cq6cq3cq6Lf,sin q5=Pz7+sq4Lu−sq4sq6Lfcq4cq6Lf,T73=nx7ox7ax7Px7ny7oy7ay7Py7nz7oz7az7Pz70001.

For the last three DOFs of the kinematic model, *q*_7_ to *q*_9_ can be obtained by the transformation matrix _9_^6^*T*:

(12) q9=a tan 2ny9,oy9,q8=a sin−ay9,q7=a tan2ax9,az9,T96=nx9ox9ax9Px9ny9oy9ay9Py9nz9oz9az9Pz90001.

The information of the upper limb's kinematic can be obtained by the transformation matrix of each joint, and the distance information can be got after static calibration. Besides, the human upper extremity movements are redundant, so there are eight solutions in *q*_3_- *q*_9_. According to the normal range of the motion of human body in Table 3, a part of the solutions can be removed. And according to the principle of minimum change of joint angle, the unique solution can be calculated.

### 2.4. Assessment Indices

The motor function of the upper extremity is presented not only in the Cartesian space but also in the joint space. In this paper, seven parameters are used to evaluate the ability of upper limb movement, which can be classified as joint space kinematic indices, Cartesian space kinematic indices, and dynamics indices.

#### 2.4.1. Joint Space Kinematic Indices

*(1) Range of Motion (ROM)*. The index measures the degree how much each joint contributes to the movement, which can be calculated by the differences between *q*_*i*_ maxima and minima *q*_*i*_, where *q*_*i*_*i*=3,4,5 …, 9 is the joint angle calculated according to the kinematic model.

*(2) Shoulder Girdle Compensation (SGC)*. The index can be calculated by the differences between *q*_1_ maxima and minima *q*_1_ and *q*_2_ maxima and *q*_2_ minima, where *q*_1_ is the shoulder girdle elevation/depression angle, and *q*_2_ is the profusion/retraction angle.

*(3) Trunk Compensation (TC)*. This describes the extent to which a patient's trunk compensates, which can be obtained by the RMSE of the displacement of the trunk.

#### 2.4.2. Cartesian Space Kinematic Indices

*(1) Aiming Angle (AA)*. This index is defined as the angular between target trajectory and the vector of the starting point to the peak speed point [27]. The higher the value, the worse the motor control. For better understanding this value, our system makes a progress on this index, as follows:

(13) $$\begin{array}{c} AA=1-\frac{AA^{*}}{2\pi }, \end{array}$$

where AA is the improved indicator value, ranging from 0 to 1 and AA^*∗*^ is the original value, ranging from 0 to 2*π*. The smaller the *AA*^*∗*^ is, the closer the AA is to 1. Thus, the closer the score is to 1, the better motor control ability the patient has.

*(2) Motion Error (ME)*.

(14) $$\begin{array}{c} R_{x}=\frac{\sqrt{1/m-1\sum_{k=1}^{m}{\left(\Delta s_{k}\right)}^{2}}}{L},\\ ME=\frac{1}{1+R_{x}}, \end{array}$$

where *m* is the number of sample points, Δ*s*_*k*_ is the vertical distance from the sample point to the task trajectory, and *L* is the length of the task trajectory. The score ranges from 0 to 1, and the closer the score is to 1, the better motor control the patient have.

*(3) Motion Length Ratio (MLR)*. This represents the length ratio between the patient's actual motion trajectory and the task trajectory. The value of the index is between 0 and 1. And the closer this value is to 1, the better motor control he or she takes to reach the goal.

#### 2.4.3. Dynamics Index

*(1) Useful Force (UF)*. The index assesses the useful work that the evaluation subject applies in the direction of the task trajectory while performing the evaluation task [15]. The maximum useful force is obtained when the direction of the force is the same as the direction of the task trajectory. When the angle between the direction of force and the direction of the trajectory is *π*/2, the minimum useful force is 0, which can be obtained by the following equation:

(15) $$\begin{array}{c} UF=\frac{\sum_{i=1}^{n}\overset{⟶}{F_{i}\cdot \overset{⟶}{X_{i}}}}{n},\;\left(i=1,2,\ldots ,n\right), \end{array}$$

where $\overset{⟶}{F_{i}}$ is the force vector, $\overset{⟶}{X_{i}}$ is the unit vector in the theoretical direction of movement, and *n* is the number of samples.

### 2.5. Linear Model

The Cartesian space kinematic indices (AA, ME, and MLR) describe the participants' motor control ability. And these indices are all ranging from 0 to 1. The closer the score is to 1, the better motor control ability the patient has. To reduce repetitive parameters and better evaluate upper limb motor abilities, the linear model is applied to combine these three indicators, as follows:

(16) $$\begin{array}{c} Y=KX, \end{array}$$

where *X* is the original value of the dataset of the participants, K is the weight matrix, and Y is the output of this model. In this study, we set all the entries of K as 1/3.

## 3. Experiments and Results

### 3.1. Participants

To verify the effectiveness of the evaluation system designed in this paper, 3 healthy individuals (3 males) recruited from our university participated in the study. The participants ranged in age from 23 to 25 years (mean 23.6 years, STD 1.15 years) and ranged in height from 1.70 to 1.80 m (mean 1.76 m, STD 0.05 m). All participants are right-handed. The details are shown in Table 4.

### 3.2. Experimental Procedure

**Figure 4:**
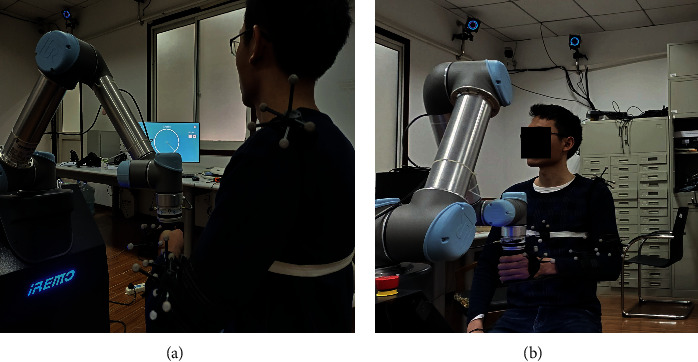
A participant who was performing the assessment.

**Figure 5:**
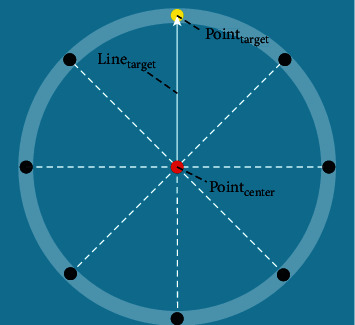
The COPTP evaluation scene. The red point is the center point, and the black points are the target points evenly distributed on the circle. The yellow point is the current destination to be reached. During the test, the subjects are asked to move back and forth between the target point and the center.

During the experiment, the robot system records the position of the end-effector in real-time, sampling at 125 HZ. Force data is measured by a 6-axis F/T sensor (Hex) at 125 HZ, and the joint space kinematics of the upper extremity are recorded with the MoCAP system at 125 HZ. Before the evaluation, participants need to be fitted with MoCAP rigid bodies and make the static calibration to establish the kinematic model. The position of the MoCAP rigid bodies fitted with is shown in Figure 4. Besides, the subjects were asked to sit in a fixed position with no restriction on trunk movement. For each assessment test, participants need to perform the center-out-point-to-point (COPTP) test in a 12 cm radius circle, as shown in Figure 5.

During the whole evaluation process, the subjects were asked not to have shoulder girdle compensation and trunk compensation as far as possible, and they need to repeat three active motion tasks with their left and right hands individually. Besides, they should guarantee the movement speed while guaranteeing accuracy. During the evaluation process, the robot and the MoCAP system communicate with each other in real-time through TCP and a visual interface designed in the Unity game engine.

### 3.3. Results

All the data were processed by MATLAB and were processed by 4-order Butterworth low-pass filter. Meanwhile, the average value of 3 experiments was taken as the final result. And the Cartesian space kinematic indices were applied to the linear model to get a new index, called motor control ability (MCA). The detailed information of each participant is presented in Table 5. Figure 5 shows a healthy subject fitted with the MoCAP rigid bodies making an assessment experiment. In front of the subject was a visual-guided display of the assessment scenario.

**Figure 6:**
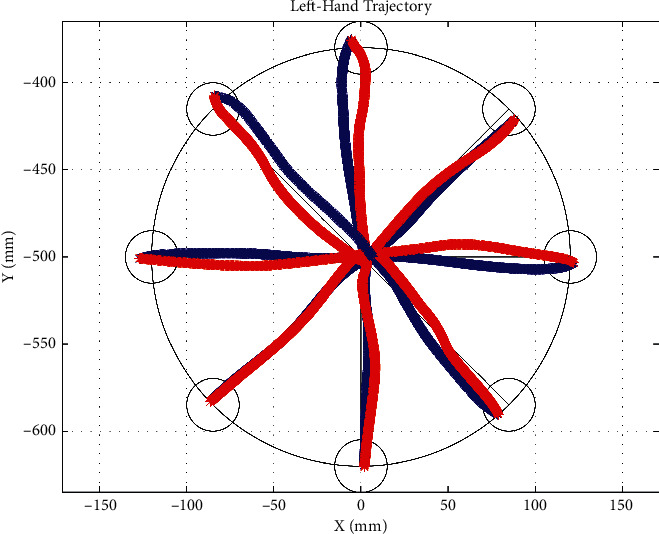
The trajectory of the right-hand motion during the COPTP task.

**Figure 7:**
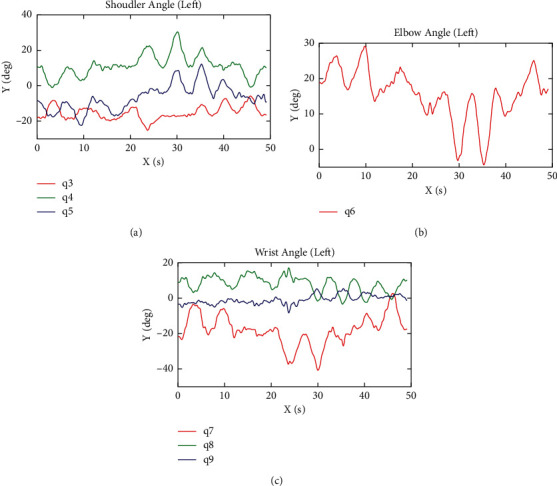
The movement trajectories of each joints' angles during the COPTP task. (a) The trajectories of shoulder joint. (b) The trajectory of elbow joint. (c) The trajectories of the wrist joint.

**Figure 8:**
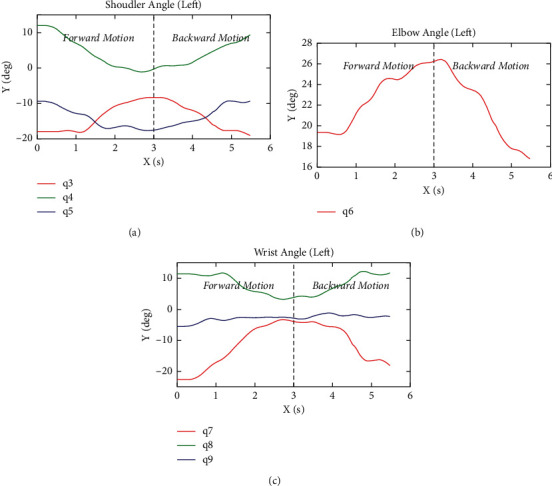
The changing traces of each joints' angles in a round-trip movement between the center point and the target point. (a) The trajectories of the shoulder joint. (b) The trajectory of the elbow joint. (c) The trajectories of wrist joint.

In this paper, we take a right-hand test of one participant as an example and show the movement track of the participant's hand and the movement trajectories of each joint angle of the upper limb.  Figure 6 shows the trajectory of the right-hand motion during a COPTP task. The blue line shows the forward motion of leaving the center of the circle. The red line shows the backward motion of moving towards the center of the circle. And when the actual position of the end of the subject is less than the threshold value (0.2 cm), and the terminal velocity is less than the threshold value (5% of the maximum velocity in the course of the movement), the next target point and the guidance line will appear. The black line is the target trajectory.  Figure 7 shows the change trajectory of the angle of each joint of the upper limb during the assessment.  Figure 7(a) is the change curve of the shoulder joint angles. The red line represents the flexion/extension angle (*q*_3_). The green line represents the abduction/adduction angle (*q*_4_). The blue line represents the internal/external rotation angle (*q*_5_).  Figure 7(b) shows the change curve of the elbow joint angle with one-degree-of-freedom extension/flexion (*q*_6_).  Figure 7(c) is the change trajectory of the wrist joint angles. The red line represents the adduction/abduction angle (*q*_7_). The green line is the flexion/extension angle (*q*_8_). And the blue line is the ulnar/radial angle (*q*_9_). All these angles are obtained by the method mentioned above. Besides, a round-trip movement between the center point and the target point is shown in Figure 8.

## 4. Discussion

According to the experimental results in Table 5, for the shoulder joint, the mean value of ROM (*q*_4_) was the smallest of the shoulder joint, which was 18.99 degrees, and the average ROM (*q*_5_) was the biggest, which is 39.03 degrees. The result shows that the designed task of our evaluation system required more movement on the shoulder internal/external rotation. For the elbow joint, the mean ROM (*q*_6_) is the largest of all the DOFs of the upper limb, with a mean value of 40.64 degrees. This is because the movement of the elbow joint is mainly used to change the position of the hand. It also proves that this system can evaluate the elbow joint motion ability well. However, the movement of the wrist joint contributes less to change the position of the hand and more to the posture of the hand. It can be seen from the result that the mean ROM (*q*_8_) is the biggest value of the wrist joint, which reaches 31.22 degrees. And the mean ROM (*q*_9_) reaches 16.08 degrees, which is the smallest value of the wrist joint. In addition, trunk compensation and shoulder girdle compensation can also be captured to distinguish whether a patient's increase in the performance of the assessment is due to abnormal compensation or the patient's actual recovery in motor ability.

**Figure 9:**
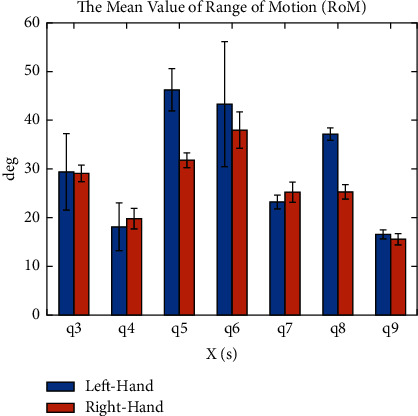
The average ROMs of the upper limb joints.

As shown in Figure 9, the result shows the comparison of mean ROM between the handedness and the nonhandedness. For the ROM of the shoulder and the wrist joint, which contributes major efforts to change the position of the hand, the ROM (*q*_3_), ROM (*q*_5_) and ROM (*q*_6_) of the nonhandedness are bigger than those of the handedness, and the ROM (*q*_4_) is less than that of handedness. The reason is that the motor function of the handedness is better than the nonhandedness, so that the nonhandedness paid more effort to complete the same COPTP task. For the ROM of the wrist joint, which contributes major efforts to change the pose of the hand, the ROM (*q*_7_) and ROM (*q*_9_) are similar and relatively smaller among all the ROM of the upper extremity motion, because the assessment scenario of our system is planar, and the rotational DOFs of the end effector are almost limited that only one rotational DOF along the vertical axis is free. In addition, the ROM (*q*_8_) of the nonhandedness is larger than that of the handedness. Since the flexion/extension angle (*q*_8_) of wrist joint can change the position of the hand in a small range, it can also prove that the nonhandedness made more inefficient works.

**Figure 10:**
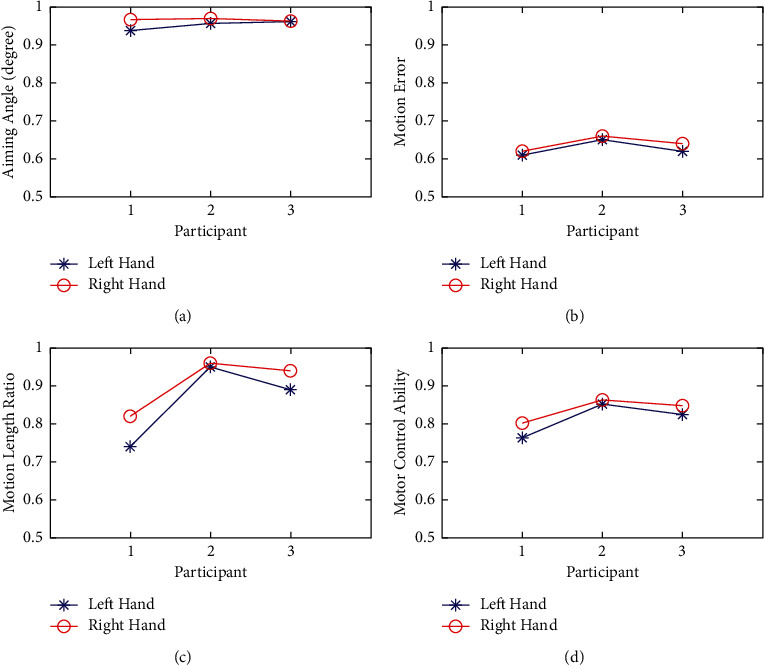
The comparison of the indices of the Cartesian space kinematics between the handedness and the nonhandedness.

For the indices of the Cartesian space kinematics, all three participants showed that the handedness performed better than the nonhandedness, which means that the handedness has a little stronger motor control than the nonhandedness, as shown in Figure 10. The aiming angle (AA), the motion error (ME), the motion length ratio (MLR), and motor control ability (MCA) of the nonhandedness are smaller than those of the handedness, which means that the motion control of the handedness is better. In this study, the weight matrix's entries were set as 1/3, for we take the same weights. The weight matrix can be different according to the focus. If aiming angle (AA) is considered more important, its weight ratio can be increased accordingly, and vice versa. Besides, the useful force (UF) of the handedness is bigger than the nonhandedness, which means that the handedness hand performs more efficiently.

In addition, the system can also obtain the change of the patient's joint angle and the position of the hand in real-time. As shown in Figure 6, it can be seen visually that the subjects are exactly moving along the target trajectory, with the right-angled 45-degree trajectory being the most accurate. According to Figure 7, each DOF presents the periodic change, and the different movement direction corresponding to the change of joint angle is different. As shown in Figure 8, during the procedure of this round-trip movement, each joint movement shows the characteristics of reciprocating movement that firstly increased and then decreased or firstly decreased and then increased. During this reciprocating motion, the movement of the shoulder and elbow joint angles changed more than the wrist joint, because the shoulder and elbow joint contribute greater efforts to change the position of the hand. Besides, the angle of wrist adduction/abduction angle *q*_7_ changed more, but the other angles of the wrist joint changed less in this reciprocating motion.

As can be seen from Table 6, our evaluation system is more comprehensive than other similar evaluation systems that includes joint space kinematics, Cartesian space kinematics, and dynamic space assessment. Our system provides more in-depth and comprehensive insights for the evaluation of patient's upper limb motor ability and can identify which specific joint's movement of the patient's upper limb is different from that of a healthy person, so that the clinician can make a specific recovery training for patients. In addition, as well as the trunk compensation, the compensation of the shoulder girdle can be detected, which can distinguish whether a patient's improvement is based on recovery or abnormal compensation, thus making a more accurate analysis for the next stage of rehabilitation planning.

## 5. Conclusion and Limitation

In this paper, we proposed a novel assessment system for evaluating the motor function of the human upper extremity. And a 9-DOF kinematic model is built, so that all the movements of the upper limb's joints can be caught in real-time, which can be used to figure out which joint contributes more or less to this movement. , butFurthermore, this model considers the movement of the shoulder girdle to more accurately determine the origin of the shoulder joint and can identify the compensation during the movement. After the evaluation, the assessment indices can quantitively describe the motor ability of the participants, including the movement of each joint, compensation, motor control ability, and motion efficiency. The preliminary results show that all the participants' handedness performs a little better than nonhandedness and can prove the effectiveness of our evaluation system. In addition, to complete the assessment of our evaluation system, our system requires more movements of the elbow joint. Although a horizontal evaluation scenario is selected in our system, all the functions will not be affected for any spatial scenario.

As a pilot study, the limitations of this paper are that only a simple horizontal scenario, COPTP, has been selected. However, the evaluation system can also be based on tasks such as drinking water or finger-to-nose, or 3D scenarios. Besides, we only recruited several healthy young volunteers. In the next step, we will design more complex assessment tasks combining VR to analyze the effectiveness of our evaluation system. In addition, we will recruit elderly volunteers and patients to further demonstrate the ability of the system to assess upper limb motor function.

## Figures and Tables

**Table 1:** Abbreviations.

Abbreviation	Description
SC	Sternoclavicular
AC	Acromion
EL	Lateral condyle
EM	Medial condyle
RS	Radial styloid process
US	Ulnar styloid process
MP	Metacarpal and phalangeal bone
ROM	Range of motion
SGC	Shoulder girdle compensation
TC	Trunk compensation
AA	Aiming angle
ME	Motion error
MLR	Motion length ratio
UF	Useful force
MCA	Motor control ability
MoCAP	Motion capture

**Table 2:** The origin and coordinate system of human upper limb joints.

	Thorax
*O*_*t*_	The origin coincident with SC
*X*_*t*_	The common line perpendicular to the *Y*_*t*_-axis and *Z*_*t*_-axis, pointing forward.
*Y*_*t*_	Pointing upward.
*Z*_*t*_	The line connecting SC and AC, pointing to AC.

	Shoulder
*O*_*s*_	The origin coincident with AC.
*X*_*s*_	The line perpendicular to the plane formed by EL, EM, and AC, pointing forward.
*Y*_*s*_	The line connecting *O*_*s*_ and the midpoint between EL and EM, pointing proximally.
*Z*_*s*_	The common line perpendicular to the *Y*_*s*_-axis and *X*_*s*_-axis, pointing right.

	Elbow
*O*_*e*_	The midpoint between EL and EM.
*X*_*e*_	The line perpendicular to the plane formed by EL, EM, and O_s_, pointing forward.
*Y*_*e*_	The line connecting *O*_*e*_ and *O*_*s*_, pointing upward.
*Z*_*e*_	The common line perpendicular to the *X*_*e*_- axis and *Y*_*e*_-axis, pointing right.

	Wrist
*O*_*w*_	The midpoint between RS and US.
*X*_*w*_	The common line perpendicular to the *Z*_*w*_-axis, and *Y*_*w*_-axis.
*Y*_*w*_	The line connecting *O*_*e*_ and *O*_*w*_, pointing proximally.
*Z*_*w*_	The line perpendicular to the plane through the US, RS, and the midpoint between EL and EM.

	Hand
*O*_*h*_	The origin is coincident with MP.
*X*_*h*_	Same as *X*_*w*_-axis.
*c*	Same as *Y*_*w*_-axis.
*c*	Same as *Z*_*w*_-axis.

**Table 3:** DH parameters for kinematic model of human right limb.

Frame	*θ*_*i*_	*α*_*i*_	*a*_*i*_	*d*_*i*_	Motion range (deg)
3	*q*_3_+*π*/2	0	0	0	−60∼155
4	*q*_4_+*π*/2	*π*/2	0	0	−150∼35
5	*q*_5_ − *π*/2	−*π*/2	0	*d*_3_(*L*_*u*_)	−80∼60
6	*q*_6_+*π*/2	−*π*/2	0	0	−90∼70
7	*q*_7_+*π*/2	*π*/2	0	*d*_5_(*L*_*f*_)	−95∼85
8	*q*_8_+*π*/2	*π*/2	0	0	−35∼65
9	*q*_9_+*π*/2	*π*/2	*a*_7_(*L*_*h*_)	0	−35∼20

**Table 4:** Participants characteristics.

Subject	Age	Gender	Handedness	Height (m)	Weight (kg)	BMI
1	25	Male	Right	1.7	60	20.76
2	23	Male	Right	1.8	66	20.37
3	23	Male	Right	1.8	76	23.45

**Table 5:** The participants' detailed datum of the evaluation indices.

Evaluation indices	Participant 1		Participant 2		Participant 3		Mean value
	Left	Right	Left	Right	Left	Right	
ROM (*q*_3_) (deg)	20.61	34.75	36.30	31.31	31.31	21.25	29.26
ROM (*q*_4_) (deg)	13.77	18.97	23.65	23.17	17.07	17.30	18.99
ROM (*q*_5_) (deg)	48.07	35.85	56.73	32.79	33.97	26.77	39.03
ROM (*q*_6_) (deg)	35.24	36.64	60.88	44.08	33.85	33.14	40.64
ROM (*q*_7_) (deg)	24.08	30.03	26.99	25.91	18.69	19.77	24.25
ROM (*q*_8_) (deg)	32.03	21.91	34.57	24.91	44.85	29.02	31.22
ROM (*q*_9_) (deg)	19.58	18.53	17.76	20.88	12.41	7.30	16.08
SGC (*q*_1_) (deg)	15.99	12.59	15.41	22.21	12.15	7.59	14.32
SGC (*q*_2_) (deg)	14.06	15.57	21.48	10..20	5.11	12.98	13.84
TC (mm)	177.68	176.71	183.35	178.89	166.77	177.33	176.79
AA (rad)	0.938	0.967	0.957	0.970	0.962	0.963	0.971
ME	0.61	0.62	0.65	0.66	0.62	0.64	0.63
MLR	0.74	0.82	0.95	0.96	0.89	0.94	0.88
MCA	0.763	0.802	0.852	0.863	0.824	0.848	0.827
UF (N)	6.08	8.03	7.95	11.82	6.67	7.00	7.93

**Table 6:** The comparison between the similar assessment system.

Author	Sensor	Degree of freedom	Evaluation indices	Compensation
Huang [12]	MCU	Elbow flexion elbow pronation/supination shoulder flexion/extension shoulder internal/external rotation shoulder abduction wrist ulnar/radial deviation	Joint kinematics, Cartesian kinematics	/

Murgia [17]	Vicon	Shoulder flexion/extension shoulder internal/external rotation elbow flexion/extension elbow pronation/supination wrist flexion/extension wrist radial/ulnar deviation	Joint kinematics	Trunk Girdle

Murphy [18]	ProReflex	Shoulder flexion/extension shoulder abduction/adduction elbow flexion/extension	Joint kinematics, Cartesian kinematics	/

Hebert [19]	Motion analysis	Shoulder flexion/extension shoulder abduction/adduction shoulder axial rotation elbow flexion/extension wrist flexion/extension	Joint kinematics, Cartesian kinematics	Trunk

Our	OptiTrack	Shoulder flexion/extension shoulder abduction/adduction shoulder internal/external rotation elbow flexion/extension wrist pronation/supination wrist flexion/extension wrist radial/ulnar deviation	Joint kinematics, Cartesian kinematics, dynamics indices	Trunk shoulder girdle

## Data Availability

The data used to support the findings of this study are available from the corresponding author upon request.
